# The Use of a Molybdenum Polyoxometalated Compound to Increase the Amount of Extractives from Wood Wastes

**DOI:** 10.3390/biom8030062

**Published:** 2018-07-28

**Authors:** Marisabel Mecca, Luigi Todaro, Maurizio D’Auria

**Affiliations:** 1 Dipartimento di Scienze, Università della Basilicata, Via dell’Ateneo Lucano 10, 85100 Potenza, Italy; marisabelmecca@libero.it; 2 School of Agricultural Forestry, Food, and Environmental Science, Università della Basilicata, Via dell’Ateneo Lucano 10, 85100 Potenza, Italy; luigi.todaro@unibas.it

**Keywords:** wood extracts, water extraction, phosphomolybdic acid, phenolic compounds, inositol

## Abstract

The treatment of wood wastes of *Castanea sativa* L., *Quercus frainetto*, *Larix decidua*, and *Paulownia tomentosa* S. in autoclave in the presence of micrometric crystals of H_3_PMo_12_O_40_ showed an impressive increase of the amount of extractives. The extractives were mainly constituted of insoluble compounds that were analyzed by using gas chromatography-mass spectrometry (GC-MS) after acetylation. The GC-MS analysis of the chloroform soluble fraction of the extractives obtained from *sativa* showed the presence of methyl hexadecanoate and octadecanoic acid, that of the extractives of *frainetto* showed the presence of octadecanal and some long chain hydrocarbons. *decidua* extracts showed the presence of large amounts of sesamin, while the extractives of *P tomentosa* revealed the presence of 4-hydroxy-3,5-dimethoxybenzaldehyde, 4-hydroxy-3,5-dimethoxycimmanaldehyde, and relevant amounts of long chain hydrocarbons. The insoluble fraction showed the presence of relevant amounts of several carbohydrates and, in the case of *C. sativa*, of inositol.

## 1. Introduction

Wood represents an important fraction of vegetable biomass. In recent years, we have ensured a significant increase in the use of this material in the construction and manufacture of furniture and fixtures, replacing plastic materials, considered pollutants. However, wood processing results in the production of an enormous quantity of wood wastes, that can represent an ecological problem and a stimulus to find a possible reuse of these wastes.

Recently, we reported the composition of the extracts of thermo-treated wood (alder, cedar) by using Soxhlet extraction and different solvents [1]; furthermore, we reported the composition of the extractives of cedar when the extraction was performed both in autoclave using water and by using Soxhlet, and alder by using Soxhlet where the yields of extractives were enhanced by using molybdenum catalyst [2,3].

**Figure 1:**
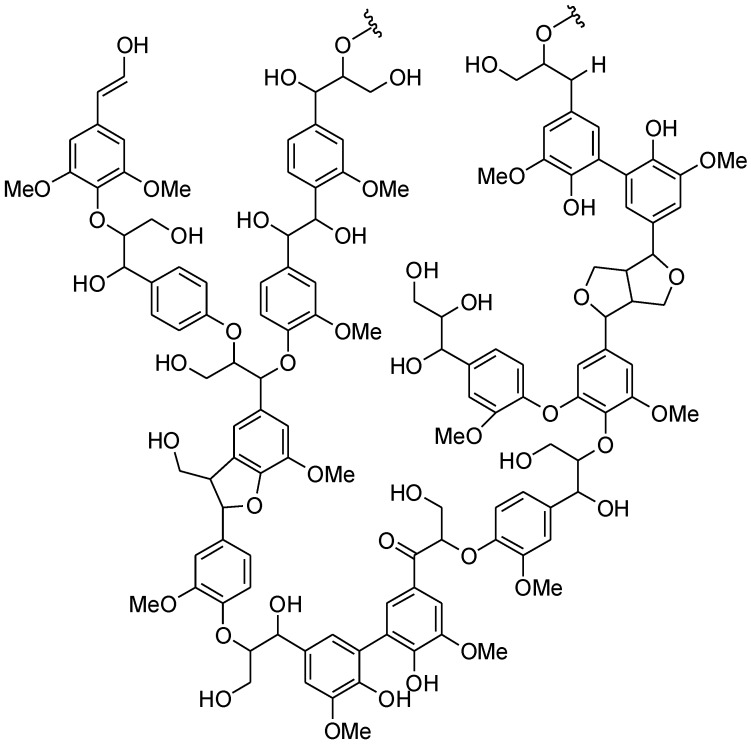
A hypothetical structure of lignin.

The possible reuse of wood wastes to obtain valuable substances is an attractive research field. Lignin accounts for 10–35% by weight of the lignocellulosic biomass, and it can represent a feedstock for aromatic chemicals (Figure 1) [4,5]. In particular, vanillin can be obtained through a lignin depolymerization process [6], and vanillin, besides aroma application, can be the starting material for the production of new polymers [7], such as epoxy polymers [8], polycarbonates [9], and polyesters [10]. In a typical alkaline oxidative lignin depolymerization vanillin is obtained in 6–12% yields from softwood and in 1–5% from hardwood [7].

Another significant chemical compound that can be obtained from wood is ferulic acid. Ferulic acid can be used as precursor of vanillin, or for its biological properties, such as antioxidant, anti-diabetic, anticancer activites, as ultraviolet (UV) absorber in cosmetic applications, or in food preservatives [11]. Furthermore, the antioxidant activity of phenolic compounds extracted from different wood has been described [12].

Recently, molybdenum containing catalysts have been used in order to reduce ester of fatty acids to alkanes [13], or to induce a benzylation reaction of arenes [14]. Furthermore, several applications showed that nanostructured catalysts can be used in order to induce selective biomass conversion [15].

In this article, we want to show the results obtained on the extracts treating thermo-treated and untreated wood with water in the presence of a claimed nanostructured molybdenum catalyst. The study was performed on *Castanea sativa* L., *Quercus frainetto*, *Larix decidua*, and *Paulownia tomentosa* S.

The extractives of *sativa* [16,17,18], *frainetto* [19], *decidua* [20,21], and *tomentosa* S. [22] has been object of specific studies in the past.

## 2. Materials and Methods

### 2.1. Sample Preparation and Heat Treatment

A total of eight sawn boards *decidua*, *tomentosa*, *sativa*, and *frainetto* with dimensions of 32 × 150 × 1000 mm (thickness × width × length, respectively), having a moisture content of 20%, were used for the experiments.

The thermo-vacuum plant facility used for the test, following the description of Ferrari [23], was a semi-industrial prototype with an internal diameter of 1.7 m, modified to perform thermo-vacuum treatments at high temperature (up to 250 °C). The cylinder walls were heated by diathermic oil circulating between double layers of steel.

The samples were dried to a moisture content of 0% in the same cylinder prior to the thermo-vacuum treatments. The drying process of the samples having an initial moisture content of 20% was carried out at 100 °C and a pressure not exceeding 25,000 Pa, corresponding to a water boiling temperature of 65 °C [16].

In the next step, samples of the abovementioned species were exposed to the thermo-vacuum treatment. Each thermo-vacuum treatment consisted of a heating phase that started at a temperature of 100 °C and reached a maximum of air temperature of 190 °C followed by a thermal treatment phase with a different constant air temperature for 2 h: 170 °C for *sativa*, 200 °C for *x decidua* and 210 °C for *tomentosa*.

Three-hour cooling phase decreased the temperature of the samples to 100 °C. The heating rate of the air was 12 °C per hour. The temperature variation of the diathermic oil, air, and wood samples was measured using thermocouples. The reason for the different treatment is that each type of wood is associated with one specific type of treatment, because of the different nature of hardwood and softwood.

### 2.2. Soxhlet Extraction

The samples of wood obtained in the sample preparation and heat treatment were dried at 105 °C overnight. Then, it was ground through a 40 mesh screen using a Wiley Mill (Thomas Scientific, Swedesboro, NJ, USA) in agreement with TAPPI T 204 cm-97 procedure.

The obtained material (1.0 g) was put into an extraction thimble, and it was put into a Soxhlet extraction apparatus. The sample was extracted with 300 mL of 1:2 ethanol/toluene mixture (*v/v*) for 7 h. After this period, the solvent was then evaporated in vacuo.

### 2.3. Lignin Content

The remaining sawdust was transferred to a 50 mL beaker, a cold H_2_SO_4_ solution (72%) (15 mL) was added, and the mixture was frequently stirred for 2 h at room temperature.

The mixture was then diluted to 3% (*w/w*) with 560 mL of distilled water, heated under reflux for 4 h, filtered, and washed with 500 mL of water. The residue was dried at 105 °C to a constant mass. The holocellulose content was determined by difference between the residue amount after extraction and the lignin content.

### 2.4. Autoclave Extraction

A sample of wood (10 g) was put into an airtight glass jar with 50 mL of distilled water and placed into the autoclave (Vapor Matic 770, Milan, Italy) for 20 min, at a temperature of 120 °C and a pressure of 1 bar for the extraction. Sample was filtered and frozen at a temperature of −28 °C. Then, it was lyophilized to remove water. The obtained mixture was fractionated as follows: the mixture was treated with chloroform (20 mL) and filtered, the solvent was evaporated, and the residue was chromatographed using tin layer chromatography on silica gel in the presence of a fluorescent indicator and using a hexane/ethyl acetate mixture. The different zones revealed by UV irradiation were separated and eluted with ethyl acetate. The solvent was evaporated, and the residue was analyzed as described in Section 2.9. The chloroform insoluble fraction was treated as described in Section 2.8.

### 2.5. Synthesis of H_3_PMo_12_O_40_ Catalyst

The heteropolyoxometalate H_3_PMo_12_O_40_ was prepared according to literature procedures. 3.4 mL of H_3_PO_4_ (85% m/m) and 142 mL of 12 M HClO_2_ were added successively to 210 mL solution of 2.85 M Na_2_MoO_4_. The warm solution took on a yellow color, and the disodium salt Na_2_H(PMo_12_O_40_) has precipitated. After cooling to room temperature, the microcrystalline powder was filtered and air-dried. The salt was then recrystallized from a (20 mL/100 mL) Et_2_O/H_2_O mixture obtaining about 50 g of a greenish precipitate. H_3_PMo_12_O_40_ was obtained from a solution of 50 g of Na_2_HPMo_12_O_40_, just recrystallized, in 100 mL of H_2_O, acidified by 25 mL of 12 M HCl, and extracted with 150 mL of Et_2_O. When water was added, yellow crystals of H_3_PMo_12_O_40_ precipitated. The solid so obtained was filtered and dried at 70–80 °C. Fourier Transform-Infrared (FT-IR) spectrum of the catalyst showed peaks at the frequency ν_max_ 750, 830, 952, and 1064 cm^−1^, in agreement with reported data. Infrared spectra were obtained utilizing a Bruker Alpha FT-IR spectrophotometer (Bruker Photonics, Billerica, MA, USA) configured for attenuated total reflectance at ambient temperature.

### 2.6. Synthesis of H_3_PMo_12_O_40_ Nanoparticles

In a typical procedure 0.3 mmol of H_3_PMo_12_O_40_·13H_2_O was dispersed in 50 mL hexane and the obtained mixture was stirred vigorously for 30 min at room temperature in order to forming a homogeneous dispersion. This dispersion was transferred into a tflon-lined stainless autoclave and filled 80% of its total volume. The autoclave was sealed and maintained at 150 °C for 12 h and then cooled to room temperature. Finally, the produced powder was filtered and dried in vacuum.

### 2.7. Autoclave Extraction in the Presence of Molybdenum catalyst

The sample (10.0 g) was put into an airtight glass jar with 50 mL of distilled water and 1 g of the catalyst and was put into the autoclave (Vapor Matic 770) for 20 min, at a temperature of 120 °C and a pressure of 1 bar. Then, the sample was filtered, frozen at a temperature of −28 °C and was lyophilized to remove water. 

### 2.8. Derivatization

About 100 mg of the chloroform insoluble fraction of the extractives, 1 mL of pyridine and 1 mL of acetic anhydride were added, and the sample was allowed to sit at room temperature for 48 h. Then, the solvent was exchanged with ethanol under reduced pressure followed by drying in vacuo. The residue was chromatographed using tin layer chromatography on silica gel in the presence of a fluorescent indicator. The different zones revealed by UV irradiation were separated and eluted with ethyl acetate. The solvent was evaporated, and the residue was analyzed as described in Section 2.9.

### 2.9. Gas Chromatographic-Mass Spectrometric Analyses 

Analyses of all the extractives obtained using all the procedures previously described were accomplished with an HP 6890 Plus gas chromatography equipped with a Phenomenex Zebron ZB-5 MS capillary column (30 m × 0.25 mm i.d. × 0.25 μm FT) (Agilent, Milan, Italy). An HP 5973 mass selective detector (Agilent, Milan, Italy) was utilized with helium at 0.8 mL/min as the carrier gas. A split injector was maintained at 250 °C, and the detector at 230 °C. The oven was held at 80 °C for 2 min, then gradually warmed, 8 °C/min, up to 250 °C and held for 10 min. Tentative identification of aroma components was based on mass spectra and NIST11 library comparison. Attention: the GC-MS data are reported as area %, and area % represents only a semi-quantitative estimation of the amounts of each compound in the mixture. A quantitative estimation of the amount of each compound can be performed only by using calibration curves for each compound; however, it is very difficult to do it for a complex mixture of compounds.

## 3. Results and Discussion

In this study a sample of thermotreated *P. tomentosa* S. was used. In this case, *Paulownia* sample was treated at 210 °C. Samples of *C. sativa* thermotreated at 170 °C and of *L. decidua* thermotreated at 200 °C were also utilized. Furthermore, *Q. frainetto* samples were available and were used. The composition in extractives (Soxhlet extraction by using ethanol/toluene mixture), lignin and holocellulose of the wood species has been reported in the Table 1.

All the samples were treated with water in autoclave. The water extracts were lyophilized and the residue was extracted in chloroform. The amount of the extractives is reported in Table 1. The extractives has been divided into two different fractions: a chloroform soluble fraction and the insoluble one. The chloroform soluble fraction (Table 1) was analyzed by using gas chromatography-mass spectrometry (GS-MS) and the results are collected in Table 2. The chloroform insoluble fraction was acetylated in pyridine and acetic anhydride, and then analyzed by using GC-MS. The results are collected in Table 3.

The chloroform soluble fraction of *C. sativa* contains mainly 5-hydroxymethylfurfural, 4-hydroxy-3,5-dimethoxybenzaldehyde, coniferyl alcohol, and 4-methoxy-4’,5’-methylenedioxybi-phenyl-2-carboxylic acid. The insoluble fraction contains mainly ribose, glucose, and inositol. The chloroform soluble fraction obtained from the extracts of *Q. frainetto* cannot identify a large number of components. The main identified compound was sesamin. It is noteworthy that, in this case, a sterol can be observed. Probably, in this case, sterols are present in wood as glycosides with some carbohydrates that render them soluble in water and not in chloroform. The insoluble fraction contains mainly ribose and mannose. The chloroform soluble fraction of the extracts of *L. decidua* contains mainly 5-hydroxymethylfurfural and 4-hydroxy-3-methoxycinnamaldehyde. The insoluble fraction showed the presence of glucose, galactose, and mannose. Finally, the thermotreated sample of *P. tomentosa* gave a chloroform soluble fraction containing mainly 3,5-dimethoxy-4-hydroxycinnamaldehyde, 4-hydroxy-3-methoxycinnamaldehyde, and 4-hydroxy-3,5-dimethoxybenzaldehyde. In the insoluble fraction mannose, iditol, and inositol were found.

**Figure 2:**
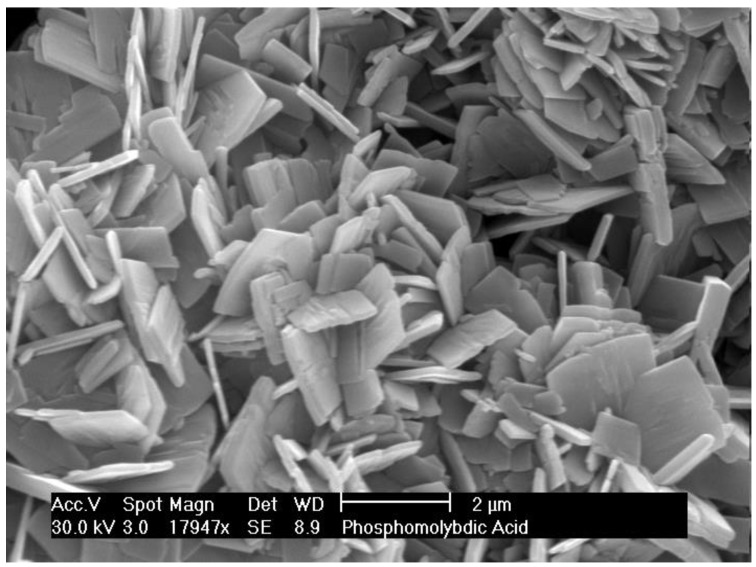
Enviromental Scanning Electron Microscopy (ESEM) of H_3_PMo_12_O_40_. Acc. V: Voltage; Magn: magnification; Det: detector; WD: wide dimension.

The wood samples were treated in autoclave in the presence of ‘nanostructured’ H_3_PMo_12_O_40_. The presence of an oxidant can induce both lignin and cellulose oxidative depolymerization, increasing the recovery of the extractives. The synthesis of this nanostructured compound is described in literature [24]. The described methodology requires an autoclave treatment of the hexane suspension of the 12-molybdophosphoric acid. However, in our hands, nanostructured material was not obtained, but we obtained only crystals with micrometric dimension (Figure 2). Probably, our difficulty to obtain nanostructured H_3_PMo_12_O_40_ is due to a lack in the experimental description of the procedure.

However, the obtained polyoxometalated compound showed an interesting behavior. The autoclave treatment of the wood samples in the presence of 10% H_3_PMo_12_O_40_ gave the results reported in Table 4. An impressive increase in the amount of the extractives was observed. The extractives of thermo-treated *C. sativa* wood increase from 7.6% until 19.9%. The extractives of *Q. frainetto* pass from 5.3% to 12.3%. Samples of thermo-treated *L. decidua* gave 1.57% extractives, while, in the presence of “nano” phosphomolybdic acid, 10.7% extractives were obtained. Finally, wood of thermo-treated *P. tomentosa* gave only 0.93% extractives, and this amount increases until 7.3% in the samples treated in the presence of polyoxometalated molybenum compound.

The GC-MS analysis of the chloroform soluble fraction of the extractives obtained from *C. sativa* showed the presence of methyl hexadecanoate and octadecanoic acid (Table 5). The chloroform soluble fraction of the extractives of *Q. frainetto* showed the presence of octadecanal and some long chain hydrocarbons (Table 5).

*Larix decidua* extracts showed the presence of large amounts of sesamin. The chloroform soluble extractives of *P. tomentosa* revealed the presence of 4-hydroxy-3,5-dimethoxybenzaldehyde, 4-hydroxy-3,5-dimethoxycimmanaldehyde, and relevant amounts of long chain hydrocarbons.

Sesamin is a noncompetitive inhibitor of Δ5-desaturase with a Ki of 155 µM without significantly influencing Δ6, Δ9, or Δ12-desaturase enzymes [25] and not influencing transcription of any enzyme [26]; in otherwise healthy postmenopausal women given 50 g sesame seeds, there have been reductions in both eicosapentanoic acid (EPA) (12%) and arachidonic acid (8%) [27]. Sesamin appears to be able to inhibit the Δ5-desaturase enzyme which results in reduced circulating levels of EPA and arachidonic acids.

Cytochrome P-4503A is involved in metabolizing tocopheprols into carboxychroman metabolites [28] which have been confirmed to be relevant to human metabolism of vitamin E and 1 µM of sesamin is able to reduce tocopherol metabolism mediated by *CYP3A* in isolated HepG2 cells.

The insoluble fractions were analyzed after acetylation, and the results are reported in Table 6. The insoluble fraction of *C. sativa* contains some carbohydrates (α-d-ribopyranose, and the corresponding methyl derivative). However, the main component was inositol, a biological active compound known as B7 vitamin, active in the control of insulin release and polycystic ovary syndrome in women [29,30].

The use of a molybdenum polyoxometalated compound depended on the evidences that molybdenum can act as catalyst in Fenton-like reactions [31,32]. However, molybdenum polyoxometalated compounds have not been used to this purpose until now [33]. Finally, it is a cheap reagent ($590 per kg) and the pretreatment of poplar with this reagent could not be expensive. 

## 4. Conclusions

The wood extractives can be a source of biological active compounds. The extractive of native wood (in the case of *Q. frainetto*) and thermotreated wood showed the presence of bioactive compounds in low quantities. This way, coniferyl aldehyde, inositol, sesamin, for example, can be found in the extracts. It is noteworthy that the water extraction procedure allowed to obtain mainly water soluble compounds while apolar or less polar lipophilic compounds cannot be extracted by using this procedure. 

When the extraction is performed in the presence of a microcrystalline polyoxometalated compound, the amount of extractives considerably increases. On the basis of the oxidant properties of the compound used in these experiments, the main degradation of lignin was expected. On the contrary, the organic solvent soluble fraction decreases with the only exception of the results obtained by using thermotreated *L. decidua* wood. Therefore, the presence of the oxidant seems to increase the degradation processes involving the cellulose fraction of wood. Probably, cellulose degradation observed in the presence of the catalyst is due to the acidic character of the solution. 

It is noteworthy that, in the organic solvent soluble fraction obtained from *L. decidua*, relevant amounts of sesamin were obtained. Furthermore, in the water soluble fraction obtained from *C. sativa* wood, inositol was obtained as the main component of the fraction.

## Figures and Tables

**Table 1:** Extractives, lignin, and holocellulose contents in wood samples.

Wood	Extractives (%)	Lignin (%)	Holocellulose (%)	Extractives in Water and Autoclave (%)	Extractives Soluble in Chloroform (%)
*Quercus frainetto*	3.7	39.6	56.7	5.3	0.7
Thermo treated	15.7	38.1	46.2	7.6	2.7
*Castanea sativa*					
*Larix decidua*	3.4	44.5	52.1	1.6	5.1
*Paulownia tomentosa*	10.2	39.5	50.3	0.9	8.5

**Table 2:** Gas chromatography-mass spectrometry (GC-MS) analysis of chloroform soluble water extractives (results presented in area %).

Compound	Retention Time (min)	Wood			
		*Castanea*	*Quercus*	*Larix*	*Paulownia*
		Area (%)			
1-(acetyloxy)-2-butanone	4.19				0.16
5-methyl-2-furancarboxyaldehyde	4.24			0.04	0.15
Benzaldehyde	4.31		0.02		
Pentanoic acid	4.36		0.02	0.06	
Limonene	5.12		0.02		
Phenol	5.45	0.05	0.02		0.20
4-oxopentanoic acid	5.50			1.55	
2-furancarboxylic acid	5.54	0.36	0.09	0.62	
Methyl 3-furancarboxylate	5.74	0.10		0.18	
2-methoxyphenol	5.83			0.24	0.60
Maltol	6.06	0.11	0.04	0.80	
5-hydroxymethtlfurfural	7.15	3.60	0.19	12.72	
Benzeneacetic acid	7.30		0.04		
Nonanoic acid	7.40		0.06		
2-acetylresorcinol	7.61			0.80	
5-acetoxymethyl-2-furaldehyde	7.78			0.72	0.22
2-methoxy-4-vinylphenol	7.89		0.05	0.22	
*Trans*-3-methyl-4-octanolide	7.96		0.04		
Piperonal	8.07				0.83
2,6-dimethylphenol	8.16	0.17	0.39	0.37	1.57
Eugenol	8.21	0.13	0.07		
Vanillin	8.54	1.56	0.38	1.18	2.47
3’,4’-(methylenedioxy)acetophenone	8.81				0.34
(2-methoxyphenoxy)acetic acid	8.83		0.05		
*Trans*-isoeugenol	8.88		0.05		
Methyl 1,3-benzodioxole-5-carboxylate	8.92				1.56
2-methoxy-4-propylphenol	8.97			0.32	
3,4-methylenedioxyphenylacetone	9.10				0.34
Apocynin	9.21	0.21			0.62
1,3-benzodioxole-5-carboxylic acid	9.31				1.84
Methyl 4-hydroxy-3-methoxybenzoate	9.41				0.36
4-hydroxy-3-methoxybenzoic acid	9.62	1.96		0.86	
2,6-dimethoxy-4-(2-propenyl)phenol	9.82	0.49			
3,4,5-trimethoxyphenol	9.85		0.16		
4-hydroxy-3,5-dimethoxybenzaldehyde	10.23	4.06	1.49	2.30	4.48
3-(4-hydroxy-3-methoxyphenyl)-2-propenal	10.66	1.84		5.16	6.61
Coniferyl alcohol	10.72	4.73			
1-(2,4,6-trihydroxyphenyl)-2-pentanone	10.97				3.62
4-hydroxy-3,5-dimethoxybenzoic acid	11.07	2.26	0.75		
Ethyl 4-hydroxy-3-methoxybenzoate	11.17	1.87			
Hexadecanoic acid	11.74	2.12		0.87	0.85
Methyl 3,4-dimethoxymandelate	11.92	1.23	0.58		
3,5-dimethoxy-4-hydroxycinnamaldehyde	11.99	2.14	0.69		15.89
Octadecanoic acid	12.92	1.82	0.20	0.78	
Octadecane	16.18	0.55	0.07	0.43	
Sesamin	17.22		4.68		
4-methoxy-4’,5’-methylenedioxybiphenyl-2-carboxylic acid	19.24	3.20			
Eicosane	19.79	0.32	0.19	0.48	
Methyl 3-(1-formyl-3,4-methylenedioxy)benzoate	23.92		0.65		
(1-ethyl-2-benzimidazolyl)-(1-naphthyl)methanol	26.73		10.12		
β-Sitosterol	30.73		0.16		

**Table 3:** GC-MS analysis of chloroform insoluble water extractives after acetylation (results presented in area %).

Compound	Retention Time (min)	Wood			
		*Castanea*	*Quercus*	*Larix*	*Paulownia*
		Area (%)			
Furfural	3.25		0.26		
3-furaldehyde	3.30			0.10	
5-acetoxymethyl-2-furaldehyde	7.79			1.33	
1-tetradecene	8.37				0.43
Vanillin acetate	9.41	0.45		0.82	
1,2-benzodioxole-5-carboxylic acid	9.55				0.28
3-acetoxy-4-methoxybenzaldehyde	9.77				0.90
4-acetoxy-3,5-dimethoxybenzaldehyde	10.56	0.68			1.68
Triacetyl-d-mannosan	10.67	2.63			
d-ribonolactone triacetate	10.75				1.07
Acetyl 2,3,4-tri-*O*-acetyl-β-d-xylopyranoside	10.90				1.64
β-d-ribopyranose tetraacetate	10.93	3.19	2.78		7.27
1,2,3,5-Tetra-*O*-acetyl-β-d-ribofuranose	11.01	2.34	3.26		6.87
β-d-deoxyribopyranose tetraacetate	11.04	0.56			6.28
Methyl β-d-arabinopyranoside triacetate	11.09	1.46			
Lyxopyranose tetraacetate	11.14		8.60		
Iditol hexaacetate	11.34		1.33		
α-d-xylopyranose tetraacetate	12.36			0.44	0.66
d-arabinose 2,3,4,5-tetraacetate	12.38				2.79
β-d-galactopyranose pentaacetate	12.40		2.42		
α-d-glucopyranose pentaacetate	12.45	3.12	1.96	3.45	2.35
Allo-inositol hexaacetate	12.48	5.31			
Myo-inositol hexaacetate	12.58	12.01			
d-galactofuranose pentaacetate	12.77		2.45	4.07	
Muco-inositol hexaacetate	12.90	3.62	3.35		
Scyllo-inositol hexaacetate	13.03	0.85			
β-d-galactopyranose pentaacetate	13.05			4.48	1.66
Meso-gluco-gulo-heptitol heptaacetate	13.08		1.06		
d-mannitol pentaacetate	13.10				0.54
Octadecanoic acid	13.12	1.29			
Methyl tetracosanoate	21.25	0.50			
Erucic acid	21.76	2.19			

**Table 4:** Extractives contents in wood samples after water extraction in the presence of H_3_PMo_12_O_40_.

Wood	Extractives (%)	Chloroform Soluble Fraction (%)
Thermotreated *Castanea sativa*	19.9	0.7
*Quercus frainetto*	12.3	1.2
Thermotreated *Larix deciduas*	10.7	9.7
Thermotreated *Paulownia tomentosa*	7.3	5.0

**Table 5:** GC-MS analysis of chloroform soluble water extractives in the presence of H_3_PMo_12_O_40_.

Compound	Retention Time (min)	Wood			
		*Castanea*	*Quercus*	*Larix*	*Paulownia*
		Area (%)			
Piperonal	8.41				0.21
Vanillin	8.81	0.10			1.51
1-(3-hydroxy-4-methoxyphenyl)ethanone	9.46				0.40
1,3-benzodioxol-5-carboxylic acid	9.56				2.16
1-(3,5-methylenedioxy)phenyl-1,2-propanedione	9.81				0.81
4-hydroxy-3-methoxybenzoic acid	9.94				1.06
2,4’-dihydroxy-3’-methoxyacetophenone	9.97	0.69			
4-hydroxy-3,5-dimethoxybenzaldehyde	10.51				4.13
Tetradecanoic acid	10.81	0.65	0.40		
3-(4-hydroxy-3-methoxyphenyl)-2-propenal	10.98				2.26
Pentadecanoic acid	11.45			0.17	
Methyl hexadecanoate	11.79		0.27		0.68
Hexadecanoic acid	11.88	7.30	1.92	0.81	2.54
3,5-dimethoxy-4-hydroxycinnamaldehyde	12.25	2.65			3.66
Oleic acid	13.10			0.24	
Octadecanoic acid	13.12	9.24	1.84	0.92	2.63
Octadecane	13.53		0.75		1.45
Tetradecyl benzoate	13.75		0.62		
Tricosane	14.04		1.09	0.39	0.67
Pentadecyl benzoate	14.69		0.90		
4,8,12,16-tetramethylheptadecan-4-olide	15.12				1.14
Hexadecyl benzoate	15.87		0.87		
Octadecanal	15.93		5.08	0.41	
Tetracosane	16.84		2.38	0.78	1.92
Heptadecyl benzoate	17.37		0.45		
Docosanoic acid	18.00			0.36	
Hexacosane	18.57		5.02	1.62	3.75
Sesamin	20.02			6.93	
Heptacosane	20.82		7.45	2.20	3.25
Octacosane	23.69		6.38	1.62	4.97
Squalene	24.83		0.97		0.99

**Table 6:** GC-MS analysis of chloroform insoluble water extractives in the presence of H_3_PMo_12_O_40_ after acetylation.

Compound	Retention Time (min)	Wood			
		*Castanea*	*Quercus*	*Larix*	*Paulownia*
		Area (%)			
2-Furaldehyde	3.30		0.26	0.10	
5-Acetoxymethyl-2-furaldehyde	8.19			1.33	
Piperonal	8.49				1.12
(*E*)-2-Tetradecene	8.72				0.43
Vanillin acetate	9.56	0.45		0.82	
1,3-benzodioxol-5-carboxylic acid	9.69				0.28
3-acetoxy-4-methoxybenzaldehyde	9.77				0.90
4-acetoxy-3,5-dimethoxybenzaldehyde	10.71	0.68			1.68
d-ribono-1,4-lactone triacetate	10.75				1.07
Methyl αd-ribopyranoside triacetate	10.84	2.63			
β-d-ribonopyranose tetraacetate	10.97	3.19	2.78		
β-d-deoxyribopyranose tetraacetate	11.04	0.56		1.21	6.28
Methyl β-d-arabinopyranoside triacetate	11.09	1.46			
Methyl α-d-ribopyranoside triacetate	11.11				1.45
Lyxopyranose tetraacetate	11.14		8.60		
1,2,3,5-tetra-*O*-acetyl-βd-ribofuranose	11.16	2.34	3.26		7.27
Acetyl 2,3,4-tri-*O*-acetyl-β-d-xylopyranoside	11.23				1.64
1,2,3,5-tetra-*O*-acetyl-β-d-ribofuranose	11.40			0.95	6.87
2-isoprpoxyphenyl hexanoate	12.12		10.25		
α-d-xylopyranose pentaacetate	12.24			0.44	
α-d-glucopyranose pentaacetate	12.34	3.12	1.96	8.87	2.35
2,3,4,5-tetraacetyl-d-arabinose	12.37		3.97		3.45
Allo-inositol hexaacetate	12.48	5.31			
β-d-galactopyranose pentaacetate	12.64		2.42		1.66
Myo-inositol hexaacetate	12.70	12.01	2.63		
d-galactofuranose pentaacetate	12.77		2.45	4.07	
Muco-inositol hexaacetate	12.82	3.62	0.72		
Scyllo-inositol hexaacetate	13.03	0.85			
β-d-galactopyranose pentaacetate	13.06			4.48	
d-mannitol pentaacetate	13.10				0.54
Octadecanoic acid	13.12	1.29			
Methyl octadecanoate	13.35	1.98			
Methyl tetracosanoate	21.25	0.50			
